# Supplementary material for: Novel Histologic Categorization Based on Lauren Histotypes Conveys Prognostic Information for Gastroesophageal Junction Cancers—Analysis from a Large Single Center Cohort in Germany
Source: Cancers (Basel). 2021 Mar 15;13(6):1303. doi: 10.3390/cancers13061303 (PMC8002040; doi:10.3390/cancers13061303)
Supplement: Supplementary file 1 [file cancers-13-01303-s001.pdf]

Supplementary materials

Supplementary Figure S1. Covariate Balancing of the Propensity Score matching variables (gender, localization, R-resection status). The upper figure displays the dotplot of standardized mean differences (Cohens's d) in the propensity scores and the covariates of interest before and after matching. The lower figure displays the line plot of standardized differences before and after matching.

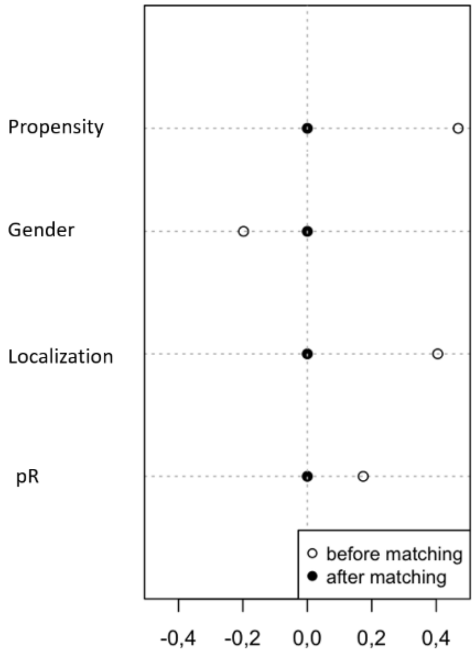

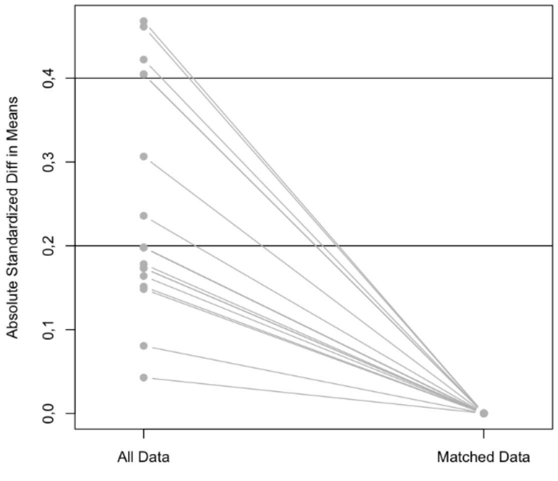

Supplemental Figure S2. Flow diagram of the patient database extraction.

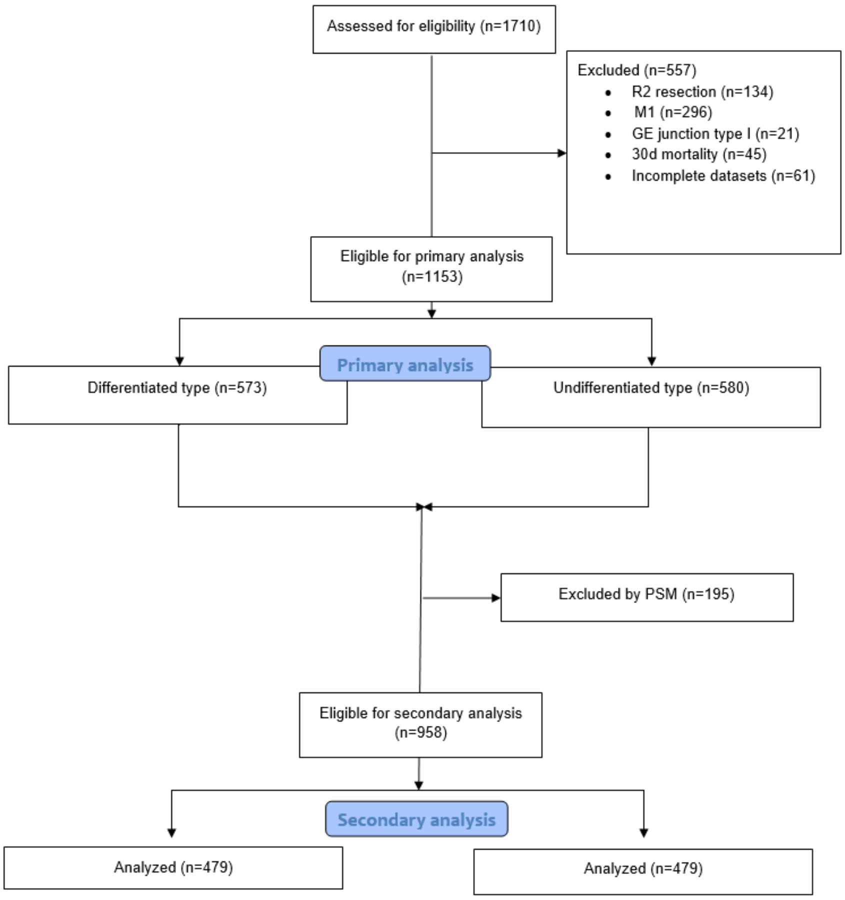

Supplemental Table S1. Propensity score balancing data.

Covariates	Means Treated		Means Control		SD Control		Std. Mean Diff.	
	**Before**	**After**	**Before**	**After**	**Before**	**After**	**Before**	**After**
Propensity score	0.53	0.50	0.48	0.50	0.11	0.11	0.47	0.00
Female	0.27	0.18	0.18	0.18	0.38	0.39	0.20	0.00
Male	0.73	0.82	0.82	0.82	0.38	0.39	−0.20	0.00
AEG II/AEGIII	0.57	0.47	0.37	0.47	0.48	0.50	0.41	0.00
pR1	0.15	0.11	0.08	0.11	0.28	0.31	0.17	0.00
propensity, propensity	0.30	0.26	0.24	0.26	0.11	0.12	0.46	0.00
propensity, Gender (female)	0.17	0.11	0.10	0.11	0.22	0.23	0.24	0.00
propensity, Gender (male)	0.36	0.39	0.38	0.39	0.20	0.21	−0.04	0.00
propensity, AEG_type (AEGIII)	0.35	0.28	0.22	0.28	0.29	0.30	0.42	0.00
propensity, pR1	0.09	0.07	0.05	0.07	0.18	0.20	0.18	0.00
Gender (female), Gender (female)	0.27	0.18	0.18	0.18	0.38	0.39	0.20	0.00
Gender (female), AEG_type (AEGIII)	0.20	0.09	0.07	0.09	0.26	0.29	0.31	0.00
Gender (female), pR1	0.03	0.02	0.02	0.02	0.12	0.14	0.08	0.00
Gender (male), Gender (male)	0.73	0.82	0.82	0.82	0.38	0.39	−0.20	0.00
Gender (male), AEG_type (AEGIII)	0.37	0.37	0.29	0.37	0.46	0.48	0.16	0.00
Gender (male), pR1	0.12	0.09	0.07	0.09	0.25	0.28	0.15	0.00
AEG_type (AEGIII), AEG_type (AEGIII)	0.57	0.47	0.37	0.47	0.48	0.50	0.41	0.00
AEG_type (AEGIII), pR1	0.07	0.04	0.03	0.04	0.18	0.20	0.15	0.00
pR1, pR1	0.15	0.11	0.08	0.11	0.28	0.31	0.17	0.00

Supplemental Table S2. Univariate and multivariate regression analysis for OS in a PSM cohort.

UNIVARIATE	HR	CI95%	*p*-value	MULTIVARIATE	HR	CI95%	*p*-value
Age	1.017	1.010–1.025	**<0.0001**		1.019	1.011–1.027	**<0.0001**
Siewert type II	1.000				1.000		
Siewert type III	1.039	0.881–1.227	0.647		0.995	0.840–1.179	0.956
Gender (Ref: female)	1.222	0.991–1.508	0.061		1.201	0.968–1.490	0.096
Neoadjuvant CTx	0.914	0.771–1.082	0.296		0.920	0.764–1.106	0.374
Differentiated	1.000				1.000		
Undifferentiated	1.307	1.107–1.544	**0.002**		1.414	1.118–1.788	**0.004**
D2-dissection	1.153	0.964–1.378	0.118		0.860	0.713–1.037	0.114
UICC$							
UICC I	1.000				1.000		
UICC II	2.570	1.958–3.373	**<0.0001**		2.525	1.911–3.336	**<0.0001**
UICC III	5.984	4.648–7.703	**<0.0001**		6.268	4.799–8.187	**<0.0001**
pR1 (Ref.: R0)	2.435	1.917–3.093	**<0.0001**		1.456	1.133–1.871	**0.003**
Grading (G1/2 vs G3/4)	1.268	1.060–1.516	**0.009**		0.783	0.606–1.,010	0.060
Comorbidity present	1.129	0.947–1.344	0.175		1.112	0.924–1.339	0.263
Complication present*	1.271	1.056–1.529	**0.011**		1.203	0.996–1.453	0.055

Legend: HR = Hazard Ratio, CI95%: 95% Confidence Interval, * according to Clavien Dindo, $ categorical variable, first value is reference (= 1.00): UICC-stage, *p*-values printed in bold are considered statistically significant.
